# Supplementary material for: A Likelihood Approach for Real-Time Calibration of Stochastic Compartmental Epidemic Models
Source: PLoS Comput Biol. 2017 Jan 17;13(1):e1005257. doi: 10.1371/journal.pcbi.1005257 (PMC5240920; doi:10.1371/journal.pcbi.1005257)
Supplement: S1 File — (TAR.GZ) [file pcbi.1005257.s014.tar.gz › HSPH_Online-SI-Revision/PF08/n10-severe/n10-severe_PF2_table-4.pdf]

1	newly 3 weeks spec	606.	355.41	{295., 421.}	41.3515	0.236095	newly 3 weeks cumul	1634.	1011.57	{830., 1202.}	38.0924	0.212586	newly 5 weeks spec	589.	355.26	{288., 416.}	39.6842	0.223631	newly 5 weeks cumul	2883.	1726.89	{1413., 2052.}	40.1009	0.226462	newly 55 weeks cumul	5530.	5166.82	{4485., 5680.}	7.86401 0.0369904
2	newly 3 weeks spec	693.	336.33	{255., 440.}	51.4675	0.32298	newly 3 weeks cumul	1675.	926.38	{700., 1206.}	44.6937	0.265814	newly 5 weeks spec	711.	365.6	{275., 461.}	48.5795	0.296838	newly 5 weeks cumul	3118.	1647.44	{1269., 2102.}	47.1636	0.285092	newly 57 weeks cumul	5958.	5674.04	{5003., 6362.}	8.04834 0.0366421
3	newly 3 weeks spec	672.	406.14	{262., 522.}	39.7381	0.234034	newly 3 weeks cumul	1714.	1077.84	{735., 1376.}	37.3524	0.215307	newly 5 weeks spec	640.	455.67	{297., 582.}	29.4297	0.164293	newly 5 weeks cumul	3081.	1972.15	{1304., 2567.}	36.208	0.207642	newly 59 weeks cumul	4699.	6168.25	{4546., 7246.}	33.169 0.12112
4	newly 3 weeks spec	729.	297.33	{254., 347.}	59.214	0.392705	newly 3 weeks cumul	1838.	850.35	{748., 997.}	53.735	0.337156	newly 5 weeks spec	670.	302.96	{261., 354.}	54.7821	0.348025	newly 5 weeks cumul	3254.	1461.68	{1301., 1675.}	55.0805	0.349969	newly 54 weeks cumul	5574.	4850.94	{4588., 5120.}	12.972 0.0608203
5	newly 3 weeks spec	577.	333.39	{288., 398.}	42.2201	0.241893	newly 3 weeks cumul	1549.	959.68	{841., 1097.}	38.0452	0.210816	newly 5 weeks spec	572.	338.13	{298., 400.}	40.8864	0.231016	newly 5 weeks cumul	2731.	1638.89	{1455., 1853.}	39.9894	0.224266	newly 54 weeks cumul	5177.	5059.97	{4672., 5467.}	5.23141 0.0233651
6	newly 3 weeks spec	667.	291.43	{241., 354.}	56.3073	0.365221	newly 3 weeks cumul	1499.	818.15	{684., 1014.}	45.4203	0.26756	newly 5 weeks spec	615.	308.84	{257., 375.}	49.7821	0.303454	newly 5 weeks cumul	2818.	1431.	{1210., 1726.}	49.2193	0.298559	newly 55 weeks cumul	4913.	5024.68	{4657., 5559.}	5.68207 0.0244263
7	newly 3 weeks spec	404.	377.11	{296., 459.}	14.4876	0.0673291	newly 3 weeks cumul	1214.	1116.61	{856., 1367.}	14.9728	0.0704745	newly 5 weeks spec	360.	358.14	{284., 431.}	12.8889	0.0565614	newly 5 weeks cumul	2017.	1849.39	{1464., 2209.}	14.2186	0.0668916	newly 54 weeks cumul	3462.	4894.77	{3760., 5392.}	42.2938 0.150683
8	newly 3 weeks spec	687.	398.74	{296., 508.}	41.9592	0.246724	newly 3 weeks cumul	1740.	1089.24	{809., 1372.}	37.4	0.212728	newly 5 weeks spec	686.	423.9	{311., 531.}	38.207	0.217907	newly 5 weeks cumul	3148.	1931.91	{1430., 2430.}	38.6306	0.220655	newly 58 weeks cumul	4943.	5831.38	{4706., 6621.}	20.4653 0.0794377
9	newly 3 weeks spec	655.	256.7	{117., 445.}	60.8824	0.457789	newly 3 weeks cumul	1488.	641.98	{309., 1128.}	57.1035	0.410364	newly 5 weeks spec	787.	342.22	{149., 648.}	56.8844	0.417795	newly 5 weeks cumul	3066.	1289.27	{608., 2294.}	57.9925	0.425118	newly 62 weeks cumul	5852.	6152.66	{4433., 7792.}	19.8483 0.0852204
10	newly 3 weeks spec	791.	639.39	{315., 870.}	23.3742	0.136042	newly 3 weeks cumul	2024.	1596.91	{848., 2138.}	24.1487	0.13907	newly 5 weeks spec	740.	697.44	{347., 915.}	22.1108	0.113185	newly 5 weeks cumul	3581.	2990.3	{1531., 3907.}	21.5956	0.122514	newly 62 weeks cumul	5507.	7022.75	{4710., 8478.}	32.4727 0.121336
11	newly 3 weeks spec	764.	299.11	{169., 407.}	60.8495	0.433126	newly 3 weeks cumul	1667.	760.59	{474., 988.}	54.5201	0.363311	newly 5 weeks spec	893.	379.75	{225., 524.}	57.4748	0.394956	newly 5 weeks cumul	3478.	1484.81	{892., 1899.}	57.3085	0.391724	newly 61 weeks cumul	4839.	6489.79	{5215., 7490.}	34.3747 0.123619
12	newly 3 weeks spec	676.	297.61	{173., 435.}	55.9749	0.381548	newly 3 weeks cumul	1411.	752.46	{456., 1057.}	46.9142	0.297273	newly 5 weeks spec	750.	372.37	{196., 519.}	50.4467	0.330511	newly 5 weeks cumul	2924.	1464.57	{815., 2071.}	50.0188	0.324911	newly 61 weeks cumul	5377.	6389.71	{5303., 7366.}	20.2364 0.0775275
13	newly 3 weeks spec	785.	343.7	{238., 479.}	56.2166	0.374897	newly 3 weeks cumul	1843.	911.16	{626., 1236.}	50.561	0.321065	newly 5 weeks spec	871.	392.32	{275., 520.}	54.9575	0.359986	newly 5 weeks cumul	3619.	1676.1	{1169., 2225.}	53.6861	0.348527	newly 58 weeks cumul	6232.	6186.78	{5453., 6895.}	8.12677 0.0358082
14	newly 3 weeks spec	790.	431.73	{257., 658.}	45.3506	0.285935	newly 3 weeks cumul	2006.	1132.29	{712., 1690.}	43.6206	0.269777	newly 5 weeks spec	648.	480.44	{288., 689.}	28.7253	0.162544	newly 5 weeks cumul	3522.	2077.87	{1317., 3107.}	41.1116	0.250506	newly 60 weeks cumul	4964.	6380.02	{5079., 7442.}	30.0757 0.111101
15	newly 3 weeks spec	973.	446.1	{135., 831.}	54.9414	0.422883	newly 3 weeks cumul	2131.	1053.	{343., 1900.}	51.9202	0.383331	newly 5 weeks spec	967.	568.31	{203., 958.}	42.8945	0.307759	newly 5 weeks cumul	4148.	2144.73	{740., 3790.}	49.3132	0.36184	newly 64 weeks cumul	6236.	6997.37	{5016., 8833.}	22.4748 0.0901363
16	newly 3 weeks spec	860.	740.71	{565., 951.}	18.3802	0.0929351	newly 3 weeks cumul	2325.	2006.05	{1514., 2568.}	18.5346	0.0933356	newly 5 weeks spec	767.	704.3	{585., 842.}	13.0587	0.0637065	newly 5 weeks cumul	3984.	3456.49	{2701., 4378.}	17.0098	0.0852258	newly 60 weeks cumul	5671.	7017.88	{5788., 8043.}	25.5518 0.0971625
17	newly 3 weeks spec	584.	361.16	{224., 508.}	38.1575	0.227599	newly 3 weeks cumul	1339.	945.91	{616., 1312.}	30.3428	0.171061	newly 5 weeks spec	581.	418.84	{267., 564.}	29.2633	0.166197	newly 5 weeks cumul	2523.	1758.56	{1120., 2401.}	30.9409	0.175896	newly 60 weeks cumul	5901.	6102.3	{4657., 7275.}	15.0602 0.0651009
18	newly 3 weeks spec	574.	321.38	{230., 456.}	44.0105	0.265893	newly 3 weeks cumul	1527.	883.94	{647., 1205.}	42.1126	0.250313	newly 5 weeks spec	639.	354.49	{264., 504.}	44.5243	0.269499	newly 5 weeks cumul	2806.	1581.87	{1187., 2159.}	43.6254	0.261816	newly 58 weeks cumul	5958.	5525.5	{4684., 6489.}	11.8889 0.0555138
19	newly 3 weeks spec	718.	341.09	{287., 391.}	52.4944	0.326483	newly 3 weeks cumul	1880.	979.87	{826., 1115.}	47.8793	0.285953	newly 5 weeks spec	617.	336.15	{286., 380.}	45.5186	0.266301	newly 5 weeks cumul	3238.	1658.39	{1413., 1854.}	48.7835	0.2931	newly 54 weeks cumul	5565.	4981.15	{4591., 5517.}	10.7495 0.0503235
20	newly 3 weeks spec	409.	324.36	{281., 363.}	20.8362	0.103465	newly 3 weeks cumul	1135.	927.31	{839., 1036.}	18.2987	0.089151	newly 5 weeks spec	432.	329.96	{284., 373.}	23.6204	0.119447	newly 5 weeks cumul	2027.	1585.78	{1435., 1758.}	21.7671	0.108067	newly 54 weeks cumul	5250.	5104.27	{4825., 5449.}	4.71371 0.020906
21	newly 3 weeks spec	797.	563.69	{271., 841.}	32.2823	0.196761	newly 3 weeks cumul	1935.	1405.93	{704., 2071.}	31.4227	0.187058	newly 5 weeks spec	789.	637.8	{335., 897.}	25.7795	0.14861	newly 5 weeks cumul	3576.	2668.3	{1349., 3844.}	29.1521	0.17331	newly 62 weeks cumul	5739.	6888.63	{4984., 8493.}	25.591 0.0980765
22	newly 3 weeks spec	515.	316.48	{200., 428.}	39.9301	0.236613	newly 3 weeks cumul	1274.	820.81	{547., 1105.}	37.1405	0.215296	newly 5 weeks spec	507.	379.05	{237., 532.}	28.9053	0.15972	newly 5 weeks cumul	2335.	1552.43	{987., 2111.}	35.1979	0.202258	newly 60 weeks cumul	4471.	6151.32	{4896., 7272.}	38.1163 0.135972
23	newly 3 weeks spec	509.	418.69	{303., 553.}	22.1316	0.116142	newly 3 weeks cumul	1486.	1120.98	{816., 1400.}	26.4697	0.142479	newly 5 weeks spec	479.	455.81	{322., 583.}	17.7474	0.0853023	newly 5 weeks cumul	2515.	2019.22	{1459., 2564.}	22.3936	0.118232	newly 59 weeks cumul	4951.	6142.88	{4761., 6937.}	26.3595 0.0998768
24	newly 3 weeks spec	864.	331.54	{211., 455.}	61.6273	0.433311	newly 3 weeks cumul	1807.	843.06	{578., 1123.}	53.3448	0.345667	newly 5 weeks spec	1016.	409.99	{260., 576.}	59.6467	0.413524	newly 5 weeks cumul	3920.	1630.84	{1114., 2242.}	58.3969	0.396865	newly 60 weeks cumul	6534.	6613.13	{5593., 7618.}	9.97077 0.0431549
25	newly 3 weeks spec	883.	192.51	{100., 323.}	78.1982	0.70848	newly 3 weeks cumul	1516.	467.11	{250., 769.}	69.188	0.550488	newly 5 weeks spec	1165.	273.54	{127., 507.}	76.5202	0.691078	newly 5 weeks cumul	3941.	974.65	{457., 1690.}	75.269	0.654901	newly 63 weeks cumul	5681.	6417.07	{4652., 8005.}	21.6143 0.0865409
26	newly 3 weeks spec	596.	458.19	{171., 760.}	36.7299	0.222408	newly 3 weeks cumul	1544.	1139.78	{452., 1810.}	36.0764	0.220049	newly 5 weeks spec	608.	539.6	{173., 849.}	33.8487	0.1871	newly 5 weeks cumul	2767.	2192.9	{856., 3545.}	34.5522	0.204228	newly 62 weeks cumul	4667.	6683.82	{4736., 8258.}	45.1091 0.155818
27	newly 3 weeks spec	646.	323.2	{121., 644.}	54.7957	0.401172	newly 3 weeks cumul	1397.	778.18	{301., 1365.}	50.4839	0.344466	newly 5 weeks spec	776.	431.18	{169., 853.}	49.1108	0.352308	newly 5 weeks cumul	2980.	1595.86	{636., 2902.}	50.9463	0.360595	newly 63 weeks cumul	6059.	6531.25	{4681., 8489.}	21.7103 0.0895659
28	newly 3 weeks spec	775.	348.38	{171., 554.}	55.6774	0.398565	newly 3 weeks cumul	1597.	849.82	{431., 1345.}	49.2786	0.328013	newly 5 weeks spec	804.	451.78	{235., 741.}	46.0821	0.298794	newly 5 weeks cumul	3259.	1708.61	{875., 2682.}	49.6536	0.331431	newly 62 weeks cumul	5190.	6860.18	{5061., 8329.}	33.5703 0.121252
29	newly 3 weeks spec	901.	366.27	{132., 671.}	59.6881	0.479461	newly 3 weeks cumul	1967.	849.14	{325., 1456.}	57.7875	0.447405	newly 5 weeks spec	1020.	512.41	{182., 895.}	50.1657	0.379939	newly 5 weeks cumul	4131.	1814.39	{704., 3169.}	56.3914	0.436927	newly 64 weeks cumul	5712.	7238.27	{5328., 8838.}	30.3811 0.113348
30	newly 3 weeks spec	883.	316.62	{114., 561.}	64.6342	0.533052	newly 3 weeks cumul	1653.	731.36	{293., 1319.}	57.487	0.432476	newly 5 weeks spec	1189.	452.39	{140., 825.}	61.9521	0.508307	newly 5 weeks cumul	4088.	1577.01	{565., 2802.}	61.7136	0.49431	newly 64 weeks cumul	6316.	6952.73	{4771., 8659.}	20.3938 0.0837422
31	newly 3 weeks spec	575.	513.1	{237., 773.}	28.3478	0.152832	newly 3 weeks cumul	1530.	1328.9	{693., 1979.}	27.1908	0.147757	newly 5 weeks spec	506.	550.94	{262., 783.}	34.581	0.152419	newly 5 weeks cumul	2641.	2428.93	{1190., 3577.}	27.159	0.141447	newly 61 weeks cumul	4464.	6362.12	{4170., 7946.}	45.2312 0.156341
32	newly 3 weeks spec	390.	341.98	{289., 416.}	15.4154	0.0739017	newly 3 weeks cumul	1183.	991.7	{833., 1190.}	17.574	0.0863945	newly 5 weeks spec	341.	337.79	{282., 403.}	11.6276	0.0509049	newly 5 weeks cumul	1958.	1676.04	{1421., 1978.}	16.1716	0.07857	newly 54 weeks cumul	3806.	4905.52	{4472., 5398.}	29.3857 0.110991
33	newly 3 weeks spec	499.	305.42	{257., 357.}	38.7936	0.217276	newly 3 weeks cumul	1415.	881.33	{749., 1076.}	37.7915	0.209885	newly 5 weeks spec	521.	309.98	{261., 361.}	40.5029	0.229175	newly 5 weeks cumul	2457.	1502.79	{1289., 1803.}	38.8364	0.216941	newly 54 weeks cumul	5031.	4794.91	{4522., 5176.}	6.04949 0.0271734
34	newly 3 weeks spec	526.	368.84	{297., 464.}	30.0114	0.160656	newly 3 weeks cumul	1521.	1080.16	{868., 1315.}	29.265	0.155614	newly 5 weeks spec	469.	357.28	{281., 425.}	24.3326	0.1254	newly 5 weeks cumul	2539.	1803.62	{1493., 2148.}	29.1335	0.154298	newly 54 weeks cumul	4810.	4963.07	{4510., 5409.}	7.95655 0.0346242
35	newly 3 weeks spec	688.	330.71	{189., 503.}	51.9317	0.344461	newly 3 weeks cumul	1586.	848.	{525., 1207.}	46.8209	0.29553	newly 5 weeks spec	769.	418.61	{233., 603.}	45.7334	0.289717	newly 5 weeks cumul	3166.	1647.11	{988., 2375.}	48.0047	0.306619	newly 61 weeks cumul	5296.	6503.01	{5185., 7491.}	24.4209 0.092357
36	newly 3 weeks spec	502.	381.23	{261., 492.}	25.8984	0.141076	newly 3 weeks cumul	1350.	1035.9	{720., 1321.}	25.0015	0.134608	newly 5 weeks spec	557.	410.05	{288., 534.}	26.7774	0.146619	newly 5 weeks cumul	2471.	1846.08	{1299., 2362.}	26.2202	0.142441	newly 57 weeks cumul	5430.	5930.16	{5093., 6678.}	12.5186 0.0508582
37	newly 3 weeks spec	552.	407.96	{264., 555.}	30.3514	0.168102	newly 3 weeks cumul	1345.	1065.14	{686., 1482.}	27.374	0.145404	newly 5 weeks spec	542.	468.42	{304., 630.}	21.9225	0.112306	newly 5 weeks cumul	2492.	1985.33	{1273., 2696.}	26.2059	0.138835	newly 60 weeks cumul	4762.	6473.59	{5059., 7305.}	37.3803 0.13538
38	newly 3 weeks spec	663.	356.1	{223., 492.}	46.4646	0.290834	newly 3 weeks cumul	1606.	925.25	{559., 1272.}	42.9633	0.261383	newly 5 weeks spec	672.	420.93	{268., 579.}	38.067	0.225054	newly 5 weeks cumul	3051.	1740.96	{1091., 2394.}	43.3012	0.264036	newly 60 weeks cumul	6153.	6231.14	{4689., 7267.}	13.2189 0.0583751
39	newly 3 weeks spec	517.	372.83	{277., 501.}	28.617	0.156421	newly 3 weeks cumul	1361.	1041.62	{753., 1410.}	25.1521	0.134014	newly 5 weeks spec	517.	384.05	{298., 511.}	26.5474	0.141865	newly 5 weeks cumul	2422.	1810.47	{1344., 2417.}	26.0095	0.139389	newly 56 weeks cumul	5258.	5519.17	{4677., 6443.}	10.096 0.0421042
40	newly 3 weeks spec	579.	330.96	{269., 404.}	42.8394	0.248829	newly 3 weeks cumul	1495.	919.17	{756., 1103.}	38.5171	0.216743	newly 5 weeks spec	560.	348.78	{290., 408.}	37.7893	0.21103	newly 5 weeks cumul	2700.	1612.48	{1338., 1895.}	40.2785	0.22892	newly 56 weeks cumul	5592.	5480.52	{4804., 6100.}	6.94599 0.0310536
41	newly 3 weeks spec	748.	348.9	{108., 702.}	56.3824	0.442924	newly 3 weeks cumul	1534.	791.58	{269., 1536.}	52.3559	0.383145	newly 5 weeks spec	1008.	486.11	{151., 908.}	52.6339	0.416953	newly 5 weeks cumul	3552.	1707.08	{576., 3349.}	53.8677	0.414443	newly 64 weeks cumul	5882.	7051.	{4951., 9005.}	27.0238 0.102865
42	newly 3 weeks spec	764.	293.42	{252., 343.}	61.5942	0.419237	newly 3 weeks cumul	1835.	824.74	{724., 949.}	55.055	0.350326	newly 5 weeks spec	835.	310.19	{261., 372.}	62.8515	0.433769	newly 5 weeks cumul	3509.	1437.83	{1249., 1645.}	59.0245	0.390352	newly 55 weeks cumul	5877.	5156.15	{4801., 5499.}	12.4276 0.0581773
43	newly 3 weeks spec	917.	429.93	{148., 720.}	54.036	0.402852	newly 3 weeks cumul	2107.	1028.49	{403., 1744.}	52.0346	0.375543	newly 5 weeks spec	846.	546.29	{195., 900.}	38.8121	0.268525	newly 5 weeks cumul	3927.	2078.76	{820., 3440.}	48.0957	0.342534	newly 63 weeks cumul	5868.	7047.44	{5093., 8663.}	26.3739 0.101129
44	newly 3 weeks spec	531.	348.84	{256., 465.}	34.3202	0.192378	newly 3 weeks cumul	1403.	953.84	{728., 1264.}	32.1939	0.177435	newly 5 weeks spec	507.	380.62	{302., 485.}	26.1105	0.137429	newly 5 weeks cumul	2442.	1704.2	{1304., 2237.}	30.4881	0.165626	newly 57 weeks cumul	4771.	5815.93	{5099., 6434.}	22.4278 0.0864052
45	newly 3 weeks spec	842.	421.12	{261., 569.}	49.9857	0.320181	newly 3 weeks cumul	1994.	1095.66	{700., 1513.}	45.4102	0.279287	newly 5 weeks spec	777.	482.11	{314., 654.}	38.2613	0.225545	newly 5 weeks cumul	3630.	2036.82	{1333., 2702.}	44.1052	0.269013	newly 60 weeks cumul	5942.	6414.29	{5133., 7219.}	14.6407 0.0614443
46	newly 3 weeks spec	429.	351.92	{231., 478.}	24.2424	0.126586	newly 3 weeks cumul	1207.	936.3	{647., 1188.}	25.9768	0.138564	newly 5 weeks spec	442.	397.58	{245., 527.}	22.7692	0.11338	newly 5 weeks cumul	2135.	1710.64	{1144., 2236.}	24.3138	0.128791	newly 59 weeks cumul	4374.	5857.84	{4375., 7009.}	35.3649 0.127043
47	newly 3 weeks spec	432.	399.3	{216., 614.}	29.7222	0.145834	newly 3 weeks cumul	1154.	1053.68	{603., 1619.}	29.0884	0.143627	newly 5 weeks spec	487.	439.1	{235., 657.}	27.5113	0.139674	newly 5 weeks cumul	2160.	1922.96	{1086., 2909.}	28.3759	0.143908	newly 60 weeks cumul	4498.	5875.71	{3964., 7312.}	35.4235 0.129389
48	newly 3 weeks spec	691.	303.34	{233., 398.}	56.1013	0.367562	newly 3 weeks cumul	1624.	821.46	{646., 1015.}	49.4175	0.304129	newly 5 weeks spec	702.	332.37	{260., 428.}	52.6538	0.333717	newly 5 weeks cumul	3044.	1473.83	{1168., 1826.}	51.5825	0.323194	newly 57 weeks cumul	6315.	5711.64	{5182., 6331.}	10.9394 0.0510439
49	newly 3 weeks spec	507.	363.15	{232., 485.}	29.5207	0.165719	newly 3 weeks cumul	1374.	966.9	{660., 1295.}	30.7045	0.171807	newly 5 weeks spec	548.	407.61	{261., 533.}	26.4799	0.147187	newly 5 weeks cumul	2534.	1763.82	{1193., 2292.}	30.7869	0.173236	newly 59 weeks cumul	4591.	5991.02	{4694., 6989.}	31.8663 0.116485
50	newly 3 weeks spec	1068.	666.23	{274., 1045.}	39.4036	0.263152	newly 3 weeks cumul	2428.	1595.43	{733., 2455.}	37.2096	0.240793	newly 5 weeks spec	913.	748.86	{343., 1030.}	25.115	0.147901	newly 5 weeks cumul	4440.	3081.68	{1448., 4624.}	33.4842	0.211104	newly 64 weeks cumul	6234.	7366.08	{5328., 8736.}	24.6728 0.0982225
